# Nanomaterials: paving the way for the hydrogen energy frontier

**DOI:** 10.1186/s11671-023-03949-8

**Published:** 2024-01-03

**Authors:** Lina M. Shaker, Ahmed A. Al-Amiery, Waleed K. Al-Azzawi

**Affiliations:** 1 Department of Chemical and Process Engineering, Faculty of Engineering and Built Environment, University Kebangsaan Malaysia (UKM), P.O. Box 43000, Bangi, Selangor, Malaysia; 2 Al-Farahidi University, Baghdad, 10001 Iraq

**Keywords:** Nanomaterials, Hydrogen energy, Hydrogen storage, Fuel cells, Catalysis

## Abstract

This comprehensive review explores the transformative role of nanomaterials in advancing the frontier of hydrogen energy, specifically in the realms of storage, production, and transport. Focusing on key nanomaterials like metallic nanoparticles, metal–organic frameworks, carbon nanotubes, and graphene, the article delves into their unique properties. It scrutinizes the application of nanomaterials in hydrogen storage, elucidating both challenges and advantages. The review meticulously evaluates diverse strategies employed to overcome limitations in traditional storage methods and highlights recent breakthroughs in nanomaterial-centric hydrogen storage. Additionally, the article investigates the utilization of nanomaterials to enhance hydrogen production, emphasizing their role as efficient nanocatalysts in boosting hydrogen fuel cell efficiency. It provides a comprehensive overview of various nanocatalysts and their potential applications in fuel cells. The exploration extends to the realm of hydrogen transport and delivery, specifically in storage tanks and pipelines, offering insights into the nanomaterials investigated for this purpose and recent advancements in the field. In conclusion, the review underscores the immense potential of nanomaterials in propelling the hydrogen energy frontier. It emphasizes the imperative for continued research aimed at optimizing the properties and performance of existing nanomaterials while advocating for the development of novel nanomaterials with superior attributes for hydrogen storage, production, and transport. This article serves as a roadmap, shedding light on the pivotal role nanomaterials can play in advancing the development of clean and sustainable hydrogen energy technologies.

## Introduction

**Fig. 1:**
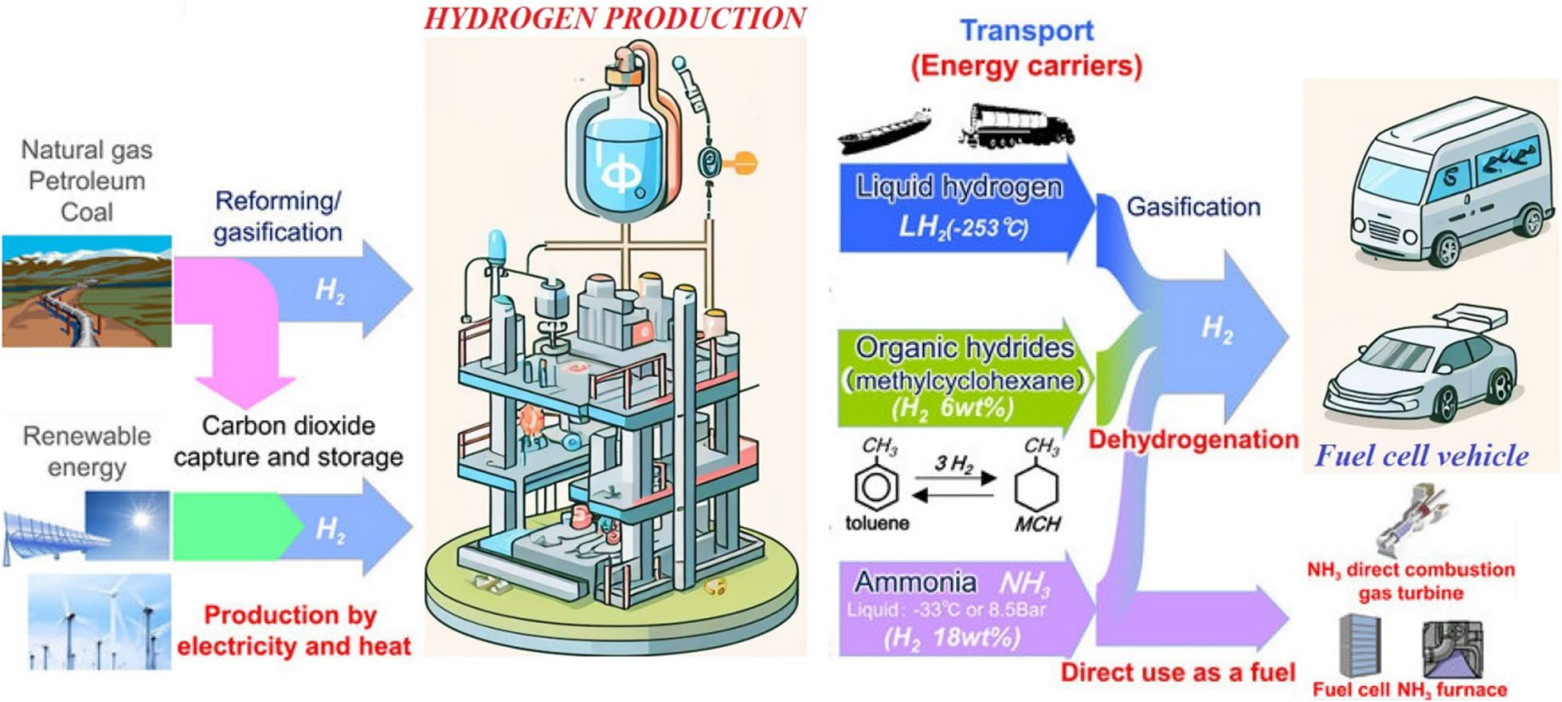
Schematic figure for different mechanisms for hydrogen production, storage, and transportation

Hydrogen energy has emerged as a promising and environmentally friendly alternative to traditional fossil fuels, owing to its high energy density and clean combustion characteristics. The versatility of hydrogen production from sources such as water, natural gas, and biomass establishes it as a sustainable energy solution (Fig. 1). However, the development of hydrogen energy technologies faces significant challenges, particularly in the realms of efficient production, storage, and transportation [1, 2]. To overcome these challenges, nanomaterials, characterized by dimensions on the nanoscale (typically less than 100 nm), have become pivotal players in the field of hydrogen energy [3–8]. The unique physical and chemical properties inherent in nanomaterials make them exceptionally well-suited for a diverse array of applications. In this context, one of the key applications of nanomaterials in hydrogen energy is their role in hydrogen storage. The inherent low density of hydrogen at standard temperature and pressure poses challenges for large-scale storage. However, nanomaterials exhibit a high surface area-to-volume ratio, enhancing their capacity for hydrogen storage. Notable examples include metal–organic frameworks (MOFs), porous materials known for their substantial hydrogen storage capacities due to expansive surface areas and hydrogen adsorption capabilities. Additionally, carbon nanotubes and graphene have garnered attention for their potential in hydrogen storage [9].

**Fig. 2:**
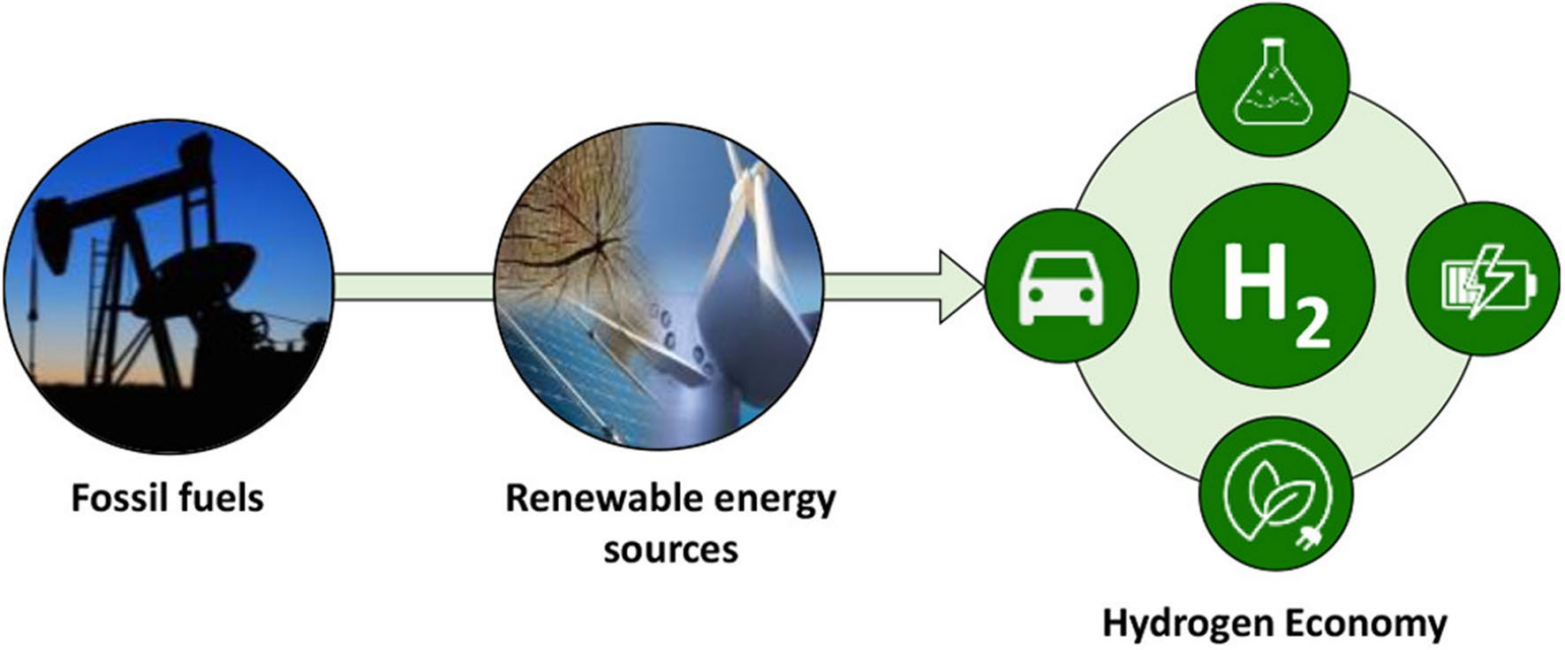
Nanomaterials in hydrogen energy [1]

Catalysis represents another crucial avenue for nanomaterials in hydrogen energy. Hydrogen fuel cells, integral to the electrochemical conversion of hydrogen into electricity, necessitate catalysts to expedite reactions. Nanomaterials, with their elevated surface areas and distinctive electronic properties, serve as effective catalysts. While platinum nanoparticles have demonstrated efficacy in hydrogen fuel cells, their cost and limited availability impede large-scale applications. Consequently, researchers explore alternative nanomaterials, such as iron, nickel, and cobalt nanoparticles, as potential catalysts for hydrogen fuel cells [10, 12]. Beyond storage and catalysis, nanomaterials are actively investigated for their role in hydrogen transport and delivery. Notably, nanomaterials are employed in improving the structural strength and durability of hydrogen storage tanks and pipelines. Composite materials incorporating carbon nanotubes and graphene exhibit promising results in enhancing hydrogen storage and transport, addressing challenges such as hydrogen embrittlement [13]. Furthermore, nanomaterials contribute to the development of highly sensitive and selective hydrogen sensors, mitigating the challenges associated with detecting the colorless and odorless nature of hydrogen gas [14, 15]. While the utilization of nanomaterials in hydrogen energy holds significant promise [16–19], overcoming challenges in scalability, cost-effectiveness, and environmentally sustainable manufacturing processes remains imperative for realizing their full potential [20]. Despite these challenges, the burgeoning field of nanomaterials in hydrogen energy, as depicted in Fig. 2, offers unprecedented opportunities for innovation and impact. Researchers worldwide are actively engaged in developing novel applications to enhance the efficiency, sustainability, and practicality of hydrogen energy technologies. Through continued research and development, nanomaterials stand poised to play a pivotal role in ushering in a clean and sustainable hydrogen energy future.

## Importance of the review article

### Comprehensive analysis of nanomaterial applications in hydrogen energy

This review article holds paramount importance in offering a thorough and contemporary examination of the pivotal role played by nanomaterials in advancing the domain of hydrogen energy. In an era marked by a global shift towards sustainable and clean energy sources, the development of hydrogen energy technologies assumes increasing significance. The distinctive properties of nanomaterials present unparalleled opportunities to enhance the efficiency, sustainability, and practicality of these technologies. Researchers, engineers, and policymakers engaged in the field of hydrogen energy will find this review article to be an invaluable resource. It delves into the current state of research on nanomaterials for hydrogen energy applications, providing a nuanced understanding of ongoing developments and shedding light on prospective directions and challenges. By underscoring the potential of nanomaterials in the realm of hydrogen energy, this review article serves as a catalyst for stimulating further research and innovation within the field.

### Potential impact on clean and sustainable hydrogen energy technologies

The significance of this review article extends to its potential impact on the landscape of clean and sustainable hydrogen energy technologies. As the global pursuit of eco-friendly energy solutions intensifies, nanomaterials emerge as critical contributors to achieving these goals. By elucidating the multifaceted applications of nanomaterials in hydrogen energy, this review article serves as a guiding beacon for future research initiatives and innovation. Moreover, this comprehensive review is poised to influence the formulation of policies and funding strategies. Policymakers can draw insights from the nuanced analysis provided herein to support and propel the advancement of nanomaterials in hydrogen energy. The review's role as an educational resource is equally significant, offering students and educators a clear and concise overview of key concepts and applications in the burgeoning field of hydrogen energy. This accessibility makes it an ideal introduction for those new to the topic, fostering a broader understanding of the potential of nanomaterials in shaping the future of clean and sustainable energy. In summation, the pivotal importance of this review article lies in its dual capacity to inform and inspire. In an era where the pursuit of sustainable and clean energy solutions is paramount, nanomaterials may well emerge as indispensable tools, and this review article serves as a guiding force, shaping and steering this crucial area of research.

## Novelty of the review article

### Unique focus on the intersection of nanomaterials and hydrogen energy

The novelty of this review article is underscored by its exclusive focus on the pivotal role of nanomaterials in advancing the domain of hydrogen energy. While substantial research exists independently on nanomaterials and hydrogen energy, this review article stands out by providing a distinctive and comprehensive examination of their intersection. One of its primary contributions lies in its in-depth discussion of various types of nanomaterials, including but not limited to carbon nanotubes, metal hydrides, and nanoporous materials. Each of these materials presents unique properties poised to enhance the efficiency and practicality of hydrogen energy technologies.

### Exploration of diverse nanomaterial applications in hydrogen energy

A key novelty of this review article lies in its exploration of the diverse applications of nanomaterials in hydrogen energy. It delves into crucial aspects such as hydrogen storage, production, and fuel cells, offering insights into the current state of research on these applications. By providing a nuanced understanding of the challenges and opportunities for further development in these domains, the article contributes to bridging existing knowledge gaps.

### Examination of environmental and economic implications

Another distinctive feature of this review article is its comprehensive examination of the potential environmental and economic benefits stemming from the integration of nanomaterials into hydrogen energy technologies. By enhancing efficiency and sustainability, nanomaterials hold the promise of reducing greenhouse gas emissions and playing a pivotal role in climate change mitigation. Additionally, the article explores the potential economic opportunities that may arise in sectors such as manufacturing and energy, emphasizing the broader impact on industry and commerce.

### Building upon existing literature and proposing new insights

This review article builds upon existing literature by synthesizing information from both nanomaterials and hydrogen energy fields. It goes beyond a mere compilation of knowledge, offering new insights and perspectives at the intersection of these diverse yet interconnected disciplines. By weaving together the disparate threads of nanomaterials and hydrogen energy, the article provides a forward-looking examination that sets the stage for future research and innovation.

In summary, the novelty of this review article resides in its ability to provide a unique and forward-thinking perspective, bringing together distinct fields and shedding light on opportunities and challenges at their intersection. By accentuating the potential of nanomaterials in hydrogen energy, this article serves as a catalyst for inspiring further research, innovation, and the development of informed policies and funding strategies.

## Historical evolution of nanomaterials in hydrogen energy

### Emergence of nanomaterials in hydrogen storage (1970s–2000s)

The intersection of nanomaterials with hydrogen energy represents a relatively recent but transformative development [21]. In the early stages, dating back to the 1970s, researchers delved into the potential of metal hydrides for hydrogen storage, laying the foundation for nanomaterial involvement [23]. Metal hydrides, capable of absorbing and releasing hydrogen, initially faced challenges such as slow kinetics. The 1990s witnessed a pivotal shift as nanomaterials, including metal hydride nanoparticles, were explored to enhance hydrogen uptake and release rates, leading to the emergence of a new generation of more efficient metal hydrides [27–29].

### Catalyst innovation in fuel cells (1990s–early 2000s)

The 1990s marked a significant period for nanomaterials in the realm of fuel cells, devices converting hydrogen and oxygen into electricity. Traditional catalysts, notably platinum, posed efficiency and durability challenges. Researchers in the 1990s and early 2000s pioneered the exploration of nanomaterials, steering away from platinum, as alternative catalysts for fuel cells. This endeavor resulted in the development of nanomaterial-based catalysts with superior efficiency and durability, thus enhancing overall fuel cell performance [33–36].

### Nanomaterials in hydrogen production (early 2000s–2010s)

In the early 2000s, nanomaterials found a new avenue in hydrogen production, a critical aspect of hydrogen energy viability. Efforts focused on improving the efficiency of methods like water electrolysis and steam methane reforming. Nanomaterials, including carbon nanotubes and metal nanoparticles, emerged as promising catalysts, contributing to significant enhancements in efficiency and sustainability [39].

### Continued growth and exploration (2010s–present)

The subsequent years witnessed continued growth and evolution in the relationship between nanomaterials and hydrogen energy. Researchers explored diverse nanomaterials and their applications, such as nanoporous materials for hydrogen storage and nanomaterial-based sensors for hydrogen detection [40]. Simultaneously, advancements in nanomaterial synthesis and characterization techniques allowed for precise control, opening new possibilities for nanoscale material manipulation [41, 42].

### Key milestones and breakthroughs

Table 1 provides a comprehensive overview of key milestones and breakthroughs in the historical sequence of the relationship between nanomaterials and hydrogen energy from 2018 to 2022. Notable achievements include the pioneering work on carbon nanotubes' hydrogen storage capacity in 1997, advancements in metal–organic frameworks (MOFs) for hydrogen storage, and breakthroughs in using nanomaterials as photocatalysts for hydrogen production from water under sunlight [48, 53, 54].

**Table 1:** Historical sequence of the relationship between nanomaterials and hydrogen energy

Year	Discovery/development	References
1975	First report on: the expression “hydrogen economy” was first proposed in the past twentieth century	[43]
1985	Smalley and co-workers at Rice University developed the chemistry of fullerenes. Fullerenes are geometric cage-like structures of carbon atoms that are composed of hexagonal and pentagonal faces. The first closed, convex structure formed was the C60 molecule	[44]
1987	First observation of carbon nanotubes by S. Iijima	[45, 46]
1995	First report on hydrogen storage in metal–organic frameworks by O. M. Yaghi et al	[47]
1997	In 1997, a groundbreaking study by A. Dillon, K. Jones, T. Bekkedahl, C. Kiang, and D. Bethune at Rice University in Texas reported the first successful storage of hydrogen in carbon nanotubes. The researchers found that the hydrogen molecules adsorbed onto the surface of the nanotubes, which provided a high surface area and a high binding energy for the hydrogen atoms. The study sparked a great deal of excitement in the scientific community, as it opened up a new avenue for hydrogen storage using nanomaterials. The researchers used a high-pressure hydrogen gas to fill the carbon nanotubes, and then measured the amount of hydrogen adsorbed using a gravimetric technique. They found that the nanotubes could store up to 4.2% hydrogen by weight, which was significantly higher than the 1–2% storage capacity of traditional materials such as activated carbon	[48]
1997	The first report on hydrogen storage in mesoporous carbon was published in 1997. In the study, the authors synthesized mesoporous carbon materials using a template method and investigated their potential for hydrogen storage. The results showed that the mesoporous carbon materials had high surface areas and pore volumes, which allowed for significant hydrogen adsorption. The study laid the foundation for further research on the use of mesoporous carbon materials as potential hydrogen storage materials	[49]
2004	The first report on hydrogen storage in single-walled carbon nanotubes was published in the journal Science in 1997 by Rodney S. Ruoff and colleagues. In the paper, the researchers described the ability of single-walled carbon nanotubes to adsorb hydrogen molecules and store them at high densities. The researchers used a combination of experimental techniques, including thermogravimetric analysis, temperature-programmed desorption, and infrared spectroscopy, to study the interaction of hydrogen molecules with single-walled carbon nanotubes. They found that at room temperature and low pressures, hydrogen molecules were physically adsorbed onto the surface of the nanotubes, forming a monolayer	[50]
2005	The first report on hydrogen storage in graphene was published in the journal Physical Review Letters in 2009 by Marco Buongiorno Nardelli and colleagues. In the paper, the researchers described the theoretical calculations of the ability of graphene to store hydrogen molecules. The researchers used first-principles calculations based on density functional theory to study the interaction of hydrogen molecules with graphene. They found that at low temperatures and high pressures, hydrogen molecules were chemically absorbed onto the surface of graphene, forming a stable bond. The researchers also found that the hydrogen storage capacity of graphene was highly dependent on the presence of defects and functional groups on the surface of the material. They proposed that the introduction of defects and functional groups could increase the surface area and create binding sites for hydrogen molecules, leading to higher hydrogen storage capacity. This discovery opened up new possibilities for the use of graphene as a hydrogen storage material for fuel cell applications. However, experimental studies are still needed to verify the theoretical predictions and to develop practical methods for synthesizing and using graphene as a hydrogen storage material	[51]
2006	The first report on hydrogen storage in iron–nitrogen-carbon nanoparticles was published in the journal Nature Materials in 2007 by Xinliang Feng and colleagues. In the paper, the researchers described the synthesis and characterization of iron–nitrogen-carbon nanoparticles and their ability to store hydrogen. The researchers synthesized the nanoparticles by pyrolyzing an iron-containing precursor in the presence of nitrogen and carbon sources. They found that the resulting nanoparticles had a core–shell structure, with an iron–nitrogen core and a carbon shell. The researchers then studied the interaction of hydrogen molecules with the nanoparticles and found that hydrogen was chemically adsorbed onto the surface of the nanoparticles, forming stable metal hydride bonds. They found that at room temperature and pressures of around 100 atmospheres, the nanoparticles could store up to 2.5% hydrogen by weight. The researchers also found that the hydrogen storage capacity of the nanoparticles was dependent on the size of the iron–nitrogen core and the thickness of the carbon shell. They proposed that optimizing the core–shell structure could lead to higher hydrogen storage capacity. This discovery opened up new possibilities for the use of iron–nitrogen-carbon nanoparticles as a hydrogen storage material for fuel cell applications. Further research is still needed to optimize the synthesis and properties of the nanoparticles for practical applications	[52]
2005	The first report on hydrogen storage in metal–organic frameworks (MOFs) with high gravimetric and volumetric capacities was published in the journal Science in 2005 by Omar Yaghi and colleagues. In the paper, the researchers described the synthesis and characterization of MOFs and their ability to store hydrogen. The researchers synthesized the MOFs by combining metal ions or clusters with organic linkers to form a porous, crystalline framework. They found that the resulting MOFs had a high surface area and could adsorb large amounts of hydrogen. The researchers then studied the interaction of hydrogen molecules with the MOFs and found that hydrogen was adsorbed onto the surface of the MOFs, forming weak van der Waals bonds. They found that at room temperature and pressures of around 100 atmospheres, some MOFs could store up to 7.5% hydrogen by weight, which is significantly higher than other materials previously studied. The researchers also found that the hydrogen storage capacity of the MOFs was dependent on the type of metal and organic linker used in their synthesis. They proposed that optimizing the composition and structure of MOFs could lead to even higher hydrogen storage capacities. This discovery opened up new possibilities for the use of MOFs as a hydrogen storage material for fuel cell applications. However, further research is still needed to optimize the synthesis and properties of MOFs for practical applications	[53]
2009	The first report on hydrogen production using metal–organic frameworks (MOFs) as photocatalysts was published in the journal Nature Materials in 2009 by Hong-Cai Zhou and colleagues. In the paper, the researchers described the synthesis and characterization of MOFs and their ability to generate hydrogen from water using sunlight The researchers synthesized the MOFs by combining metal ions or clusters with organic linkers to form a porous, crystalline framework. They found that some MOFs had the ability to absorb light and generate electron–hole pairs, which could then be used to drive the reduction of water into hydrogen and oxygen. The researchers studied the photocatalytic activity of the MOFs and found that some of them were highly efficient in generating hydrogen from water under visible light irradiation. They also found that the hydrogen production rate and efficiency could be enhanced by optimizing the composition and structure of the MOFs. This discovery opened up new possibilities for the use of MOFs as photocatalysts for hydrogen production, which could provide a clean and sustainable source of fuel. However, further research is still needed to optimize the synthesis and properties of MOFs for practical applications and to scale up the production of hydrogen using this technology	[54, 55]
2014	The first report on hydrogen production using carbon quantum dots (CQDs) as photocatalysts was published in the journal Nano Letters in 2014 by Junwang Tang and colleagues. In the paper, the researchers described the synthesis and characterization of CQDs and their ability to generate hydrogen from water using sunlight The researchers synthesized the CQDs from citric acid using a hydrothermal method. They found that the resulting CQDs had a high surface area and were able to absorb visible light and generate electron–hole pairs, which could then be used to drive the reduction of water into hydrogen and oxygen. The researchers studied the photocatalytic activity of the CQDs and found that they were highly efficient in generating hydrogen from water under visible light irradiation. They also found that the hydrogen production rate and efficiency could be enhanced by optimizing the size and surface chemistry of the CQDs. This discovery opened up new possibilities for the use of CQDs as a low-cost and efficient photocatalyst for hydrogen production. However, further research is still needed to optimize the synthesis and properties of CQDs for practical applications and to scale up the production of hydrogen using this technology	[56]
2018	The development of iron–nitrogen-carbon (Fe–N-C) nanoparticles as alternative catalysts for fuel cells began in the early 2000s. Conventional fuel cells use platinum-based catalysts, which are expensive and rare, leading to high production costs for fuel cells. This led to the search for alternative catalysts that are cheaper and more abundant. Fe–N-C nanoparticles were found to be a promising alternative catalyst for fuel cells. They are synthesized by heating a mixture of an iron precursor, a nitrogen precursor, and a carbon precursor under high temperature and nitrogen gas. The resulting Fe–N–C nanoparticles have a similar structure to platinum-based catalysts and exhibit similar catalytic activity for the oxygen reduction reaction (ORR), which is a key reaction in fuel cells. The development of Fe–N–C nanoparticles as an alternative catalyst for fuel cells has gained significant attention in recent years due to their high activity, stability, and low cost. They have shown to have similar or even better performance compared to platinum-based catalysts, making them a promising alternative for widespread adoption of fuel cells. However, further research is still needed to optimize the synthesis and properties of Fe–N-C nanoparticles for practical applications and to address challenges such as durability, scalability, and performance in real-world conditions	[57]

### Future directions and challenges

As the historical sequence unfolds, the relationship between nanomaterials and hydrogen energy continues to grow. The expanding applications and opportunities in various domains underscore the collaborative and innovative nature of this evolving relationship. Despite the advancements, challenges persist, including optimizing nanomaterial synthesis, ensuring practical applications, and addressing scalability issues. Ongoing research and development efforts aim to overcome these challenges, paving the way for nanomaterials to play a crucial role in the global transition to a sustainable and clean energy future.

## Overview of nanomaterial types

### Metallic nanoparticles

- *Definition and characteristics* Metallic nanoparticles, composed of metals like gold, silver, platinum, and palladium, exhibit unique characteristics due to their small size and high surface area-to-volume ratio. These properties include high reactivity, catalytic activity, and distinctive optical features.
- *Examples in hydrogen applications* In the realm of hydrogen energy, metallic nanoparticles, particularly platinum nanoparticles, have been extensively researched as catalysts for various reactions. For instance, platinum nanoparticles serve as catalysts in fuel cells due to their effectiveness in the oxygen reduction reaction. Ongoing research explores alternatives like palladium and nickel nanoparticles to address cost and availability concerns [58, 59].
- *Contribution to hydrogen storage and production *The distinctive properties of metallic nanoparticles make them pivotal in hydrogen-related applications. Their high surface area and reactivity are harnessed for catalyzing reactions essential to hydrogen production, storage, and fuel cell functionality. Despite the challenges posed by platinum's cost and scarcity, exploration into alternative metallic nanoparticles showcases promising avenues for sustainable hydrogen technologies.

### Metal–organic frameworks (MOFs)

- *Introduction and porous nature* Metal–Organic Frameworks (MOFs) represent a class of porous materials comprising metal ions or clusters connected by organic ligands. Their porous structure, tunable pore size, and versatile chemistry make them attractive for applications such as gas storage, separation, and catalysis.
- *Examples with high hydrogen storage capacities* In the context of hydrogen energy, MOFs have garnered attention for their potential as hydrogen storage materials. Researchers have successfully designed MOFs with high hydrogen storage capacities by carefully tuning the metal ions and ligands. This makes MOFs a promising candidate for fuel cell vehicles.
- *Addressing challenges in hydrogen storage* While MOFs exhibit great potential, challenges such as low hydrogen uptake and slow release rates must be addressed for them to become practical hydrogen storage materials. Ongoing research aims to overcome these hurdles and unlock the full potential of MOFs in advancing hydrogen energy technologies.

### Carbon nanotubes

- *Definition and structure* Carbon Nanotubes (CNTs) are cylindrical carbon structures with nanometer-scale diameters. They possess exceptional mechanical, electrical, and thermal properties, making them attractive for various applications.
- *Exploration for hydrogen storage* In the domain of hydrogen energy, CNTs have been studied both as catalysts for hydrogen production and as components of hydrogen storage materials. Researchers have developed CNT-based catalysts for processes like steam methane reforming, a common method for hydrogen production. Additionally, CNTs have been incorporated into hydrogen storage materials, such as metal hydrides, to enhance their hydrogen uptake and release kinetics [61, 62].
- *Advantages and challenges* The unique properties of CNTs contribute to their appeal, including high strength, excellent electrical and thermal conductivity, and the ability to be functionalized for specific applications. However, challenges exist, and ongoing research delves into addressing these challenges while harnessing the advantages of CNTs for advancements in hydrogen energy.

### Graphene

- *Introduction and exceptional properties* Graphene is a two-dimensional carbon material comprising a single layer of carbon atoms arranged in a hexagonal lattice. Its exceptional properties, including high conductivity, mechanical strength, and surface area, make it versatile for a wide range of applications.
- *Applications in hydrogen technologies* In the context of hydrogen energy, researchers have explored graphene's potential as a catalyst for hydrogen production and as a component of hydrogen storage materials. Examples include graphene-based catalysts for water splitting, a common method for hydrogen production from water. Additionally, graphene has been integrated into hydrogen storage materials, such as MOFs, to enhance their hydrogen storage capacities [54, 64].
- *Contributions to hydrogen storage advancements *The exceptional properties of graphene contribute significantly to advancements in hydrogen storage. Its high surface area, conductivity, and strength play a crucial role in improving the efficiency and practicality of hydrogen storage materials. Ongoing research explores diverse applications of graphene in the field of hydrogen energy.

In addition to these four types of nanomaterials, various other types have been studied in the context of hydrogen energy. For instance, nanoparticles of transition metal carbides, nitrides, and borides have been explored as alternative catalysts for fuel cells. Nanoporous materials like zeolites and other metal–organic frameworks have been investigated as alternative materials for hydrogen storage. Ongoing research continues to explore new types of nanomaterials and their applications in hydrogen energy [65–68]. Table 2 provides an overview of metallic nanoparticles, metal–organic frameworks, carbon nanotubes, and graphene, including their properties such as size, surface area, reactivity, and unique electronic and mechanical properties. Understanding these properties is important for developing new and innovative applications of nanomaterials in various fields.

**Table 2:** Overview of different types of nanomaterials and their properties

Type of nanomaterial	Properties
Metallic nanoparticles	Small size (< 100 nm), high surface area to volume ratio, high reactivity, ability to tune surface properties and chemistry, optical and electronic properties dependent on size and shape
Metal–organic frameworks	Porous materials with high surface area and tunable pore size and chemistry, ability to adsorb and store gases, high thermal and chemical stability, ability to be functionalized for specific applications
Carbon nanotubes	Cylindrical structures made of carbon atoms, high strength and stiffness, excellent electrical and thermal conductivity, high surface area, ability to be functionalized for specific applications, unique electronic and mechanical properties dependent on size and chirality
Graphene	Two-dimensional material made of a single layer of carbon atoms arranged in a hexagonal lattice, high surface area, excellent electrical and thermal conductivity, high strength and stiffness, unique electronic properties dependent on size and shape, ability to be functionalized for specific applications

## Hydrogen storage using nanomaterials

### Carbon nanotubes

- *Advantages for hydrogen storage* Carbon nanotubes (CNTs) offer a high surface area conducive to hydrogen adsorption, coupled with their lightweight and robust nature. Their capacity for functionalization further enhances their application versatility.
- *Challenges for hydrogen storage* Despite these advantages, challenges include the intricate production process and handling difficulties. CNTs exhibit limitations in storing hydrogen efficiently at ambient temperature and pressure.

### Metal–organic frameworks (MOFs)

- *Advantages for hydrogen storage* MOFs, characterized by high porosity and surface area, present a promising avenue for hydrogen storage. Their tunable pore size and chemistry enable precise customization, and they exhibit notable stability and durability.
- *Challenges for hydrogen storage* Scaling up production poses a challenge, along with the energy-intensive and costly synthesis and processing requirements. MOFs also face limitations in the range of temperature and pressure suitable for hydrogen storage.

### Graphene

- *Advantages for hydrogen storage* Graphene's high surface area facilitates efficient hydrogen adsorption, complemented by exceptional electrical and thermal conductivity. Its functionalization potential adds to its versatility in various applications.
- *Challenges for hydrogen storage* While graphene holds promise, challenges include its limited ability to store hydrogen at ambient temperature and pressure. Large-scale production with consistent properties also remains a hurdle.

### Metallic nanoparticles

- *Advantages for hydrogen storage* Metallic nanoparticles boast high reactivity and surface area, offering potential for room temperature hydrogen storage. Their tunability for specific applications adds to their appeal.
- *Challenges for hydrogen storage* Challenges include limited stability and durability, difficulties in controlling particle size and shape, and a propensity for particle agglomeration and sintering.

### Advantages and challenges across nanomaterials

- *Common advantages* All nanomaterials share the advantage of a high surface area-to-volume ratio, facilitating efficient hydrogen adsorption. The atomic-level tunability of nanomaterials enhances their hydrogen storage properties.
- *Common challenges* Stability under hydrogen storage conditions is a shared challenge, with nanomaterials being prone to agglomeration or oxidation. Cost and scalability issues for practical applications, as well as the need for novel hydrogen storage systems, pose additional challenges.

### Overcoming challenges and realizing potential

- *Addressing stability concerns* Stability challenges, including agglomeration and oxidation, necessitate advanced material engineering strategies. Research focused on stabilizing nanomaterials under varying temperature and pressure conditions is vital.
- *Tackling cost and scalability issues* Overcoming cost and scalability challenges involves refining production processes and exploring innovative synthesis techniques. Collaboration between academia and industry can expedite the development of cost-effective production methods.
- *Developing practical hydrogen storage systems* To make the most of nanomaterials for hydrogen storage, practical systems must be devised. Specifically, for metal hydrides, research should aim at developing systems compatible with the requirements of portable devices and vehicles.

In conclusion, nanomaterials possess the potential to redefine hydrogen storage and facilitate the widespread use of hydrogen as a clean energy carrier. Each type of nanomaterial contributes uniquely to enhancing hydrogen storage, with advantages such as high surface area and tunability. While challenges persist, ongoing research endeavors to address stability concerns, optimize production processes, and devise practical hydrogen storage systems. As advancements continue, nanomaterials stand poised to play a pivotal role in realizing the promise of hydrogen as a sustainable energy solution. Table 3 provides an insightful overview of the applications of nanomaterials in hydrogen storage, highlighting both their advantages and associated challenges. To offer a more comprehensive understanding, let's delve into each type of nanomaterial, elucidating their roles and incorporating examples to illustrate their impact on hydrogen storage, production, and transportation.

**Table 3:** Advantages and challenges of using nanomaterials for hydrogen storage

Nanomaterial	Advantages for hydrogen storage	Challenges for hydrogen storage
Carbon nanotubes	High Surface Area: Facilitates efficient hydrogen adsorption Lightweight and Strong: Provides structural integrity Functionalization: Tailorable for specific applications	Production Difficulty: Challenges in the synthesis and handling of carbon nanotubes Limited Storage Capacity: Difficulty storing hydrogen at ambient temperature and pressure
Graphene	High Surface Area: Facilitates efficient hydrogen adsorption Excellent Conductivity: Exhibits exceptional electrical and thermal conductivity Functionalization: Tailorable for specific applications	Limited Ambient Storage: Challenges in storing hydrogen at ambient temperature and pressure Production Challenges: Difficulty in producing large quantities of graphene with consistent properties
Metal–organic frameworks MOFs)	High Porosity and Surface Area: Enables effective hydrogen adsorption Tunable Pore Size and Chemistry: Offers flexibility in optimizing hydrogen storage Stability and Durability: Exhibits robustness for long-term hydrogen storage	Production Scaling: Difficulty in scaling up MOF production for practical applications Cost and Energy Intensive: High energy and cost requirements for synthesis and processing Temperature and Pressure Limitations: MOF storage may be limited by specific temperature and pressure ranges
Metallic nanoparticles	High Reactivity and Surface Area: Enables effective hydrogen adsorption Tunable for Specific Applications: Offers flexibility in tuning for diverse uses Potential for Room Temperature Storage: Shows promise for hydrogen storage at room temperature	Limited Stability: May lack durability over extended periods Particle Control Challenges: Difficulty in controlling particle size and shape Agglomeration and Sintering: Tendency for particles to agglomerate and sinter, impacting stability

Carbon nanotubes provide a high surface area for efficient hydrogen adsorption and offer a lightweight yet robust structure. Challenges lie in their production difficulty, including synthesis and handling issues, and limitations in storing hydrogen at ambient conditions. MOFs exhibit high porosity and surface area, tunable pore size and chemistry, and stability for effective long-term hydrogen storage. Scaling up production for practical applications poses a challenge, and there are energy-intensive and cost-related issues in synthesis and processing. Graphene's high surface area and excellent conductivity facilitate efficient hydrogen adsorption. Challenges include limited ambient storage capacity, difficulties in large-scale production, and ensuring consistent properties. Metallic nanoparticles offer high reactivity, tunability for diverse applications, and the potential for room temperature hydrogen storage. Limitations include potential stability issues over extended periods, challenges in controlling particle size and shape, and a tendency for particle agglomeration.

## Nanomaterials in hydrogen production

### Nanocatalysts for hydrogen production

Hydrogen fuel cells stand as a beacon of promise for clean energy generation, operating with only water as a byproduct. However, the efficiency and cost of hydrogen production pose challenges. Nanomaterials emerge as key players in surmounting these challenges, particularly in enhancing the efficiency of hydrogen production. A pivotal application lies in their role as catalysts for hydrogen evolution reactions (HER) and oxygen evolution reactions (OER) [76].

### Catalyst synthesis and effectiveness

Nanocatalysts, synthesized through methods like chemical reduction and solvothermal synthesis, offer advantages in HER and OER due to their high surface area, reactivity, and tunable surface chemistry [77, 78]. Platinum (Pt) nanoparticles are a prevalent choice, given their high activity for HER. Despite their efficacy, the expense and scarcity of Pt prompt exploration into alternative Pt-based nanocatalysts. Alloys such as Pt-Ni and Pt–Co exhibit superior catalytic activity and stability [79].

### Diverse nanomaterials as catalysts

Beyond Pt-based nanocatalysts, other nanomaterials come into focus. Transition metal dichalcogenides (TMDs) like molybdenum disulfide (MoS2) and carbon-based nanomaterials such as graphene and carbon nanotubes show promise as catalysts for HER and OER, leveraging their high intrinsic activity, low overpotential, and tunable surface chemistry [80].

### Photocatalytic water splitting

Nanomaterials also play a crucial role in photocatalytic water splitting, converting solar energy into chemical energy for hydrogen production. Metal oxides, sulfides, and nitrides, such as titanium dioxide (TiO2), showcase potential as photocatalysts. Modified TiO2 nanomaterials, including nitrogen-doped variants, demonstrate improved photocatalytic activity [81].

### Enhancing hydrogen fuel cells

Nanomaterials contribute significantly to improving the efficiency of hydrogen fuel cells by enhancing the surface area and reactivity of catalysts. Platinum, while common, faces challenges due to cost and limited supply. Strategies involve protective coatings, catalyst structure optimization, and the incorporation of dopants to enhance stability and activity [83].

### Bimetallic nanomaterials and nanoscale engineering

Bimetallic nanomaterials, combining two metals alloyed together, present enhanced catalytic activity and stability compared to monometallic counterparts. Platinum-nickel (Pt-Ni) and platinum-cobalt (Pt–Co) bimetallic catalysts demonstrate improved performance in fuel cell applications [84]. Nanoscale engineering, exemplified by core–shell nanocatalysts, where a core of one metal is enveloped by another or a non-metal like carbon or nitrogen, exhibits heightened activity, stability, and resistance to impurities [85].

### Nanomaterials beyond fuel cells

Nanomaterials extend their impact beyond fuel cells, contributing to the efficiency and sustainability of various hydrogen production technologies like electrolysis and photocatalysis. For instance, titanium dioxide (TiO2) and zinc oxide (ZnO) nanomaterials act as efficient photocatalysts for hydrogen production through water splitting. Their ability to absorb light energy and drive chemical reactions positions them as potential alternatives to traditional photocatalysts [81, 86].

Table 4 provides a comprehensive overview of how nanomaterials enhance hydrogen production and fuel cell efficiency. Noteworthy applications include carbon quantum dots and metal–organic frameworks for photocatalytic hydrogen production, and graphene, carbon nanotubes, and metallic nanoparticles for fortifying the durability and stability of fuel cell components. Table 4, highlights how nanomaterials can be used to improve hydrogen production and increase the efficiency of hydrogen fuel cells. Carbon quantum dots and metal–organic frameworks show promise for photocatalytic hydrogen production, while graphene, carbon nanotubes, and metallic nanoparticles have potential for improving the durability and stability of fuel cell components. By utilizing the unique properties of nanomaterials, researchers can develop more efficient and cost-effective hydrogen production and fuel cell technologies.

**Table 4:** Nanomaterials for improved hydrogen production and fuel cell efficiency

Application	Nanomaterial	Advantages
Hydrogen production	Carbon quantum dots	High efficiency for photocatalytic hydrogen production, low toxicity, low cost, ability to be synthesized in large quantities
Hydrogen production	Metal–organic frameworks	High stability and durability, ability to tune pore size and chemistry for improved photocatalytic performance, high surface area for increased catalytic activity
Fuel cells	Graphene	High electrical conductivity and surface area, ability to be functionalized for specific applications, potential for improved durability and stability of fuel cell components
Fuel cells	Carbon nanotubes	High strength and stiffness, excellent electrical conductivity, ability to be functionalized for specific applications, potential for improved durability and stability of fuel cell components
Fuel cells	Metallic nanoparticles	High reactivity and surface area for improved catalytic activity, ability to be tuned for specific applications, potential for improved durability and stability of fuel cell components

The utilization of nanomaterials in hydrogen energy applications signifies a rapidly advancing realm of research, holding immense potential for sustainable energy technologies. Although challenges persist in improving efficiency, durability, and scalability, ongoing research efforts promise a future where nanomaterials contribute significantly to the realization of clean energy.

Overall, the use of nanomaterials in hydrogen energy applications represents a rapidly growing field of research with significant potential for advancing the development of sustainable energy technologies. While there are still many challenges to overcome in terms of improving the efficiency, durability, and scalability of nanomaterial-based hydrogen energy systems, the ongoing research and development efforts in this field hold great promise for the future of clean energy.

## Nanomaterials in hydrogen transport and delivery

### Strengthening hydrogen storage tanks

Hydrogen transport and delivery, integral to the hydrogen energy infrastructure, confront the challenge of storing and transporting gas at high pressures, necessitating heavy and bulky storage tanks and pipelines [87]. The exploration of nanomaterials offers a transformative avenue for enhancing the efficiency and safety of these systems.

### Nanomaterials for hydrogen storage tanks

Metal hydrides, compounds capable of absorbing and releasing hydrogen gas, present a promising direction for improving hydrogen storage. Nanoscale metal hydrides, exemplified by magnesium hydride nanoparticles, exhibit enhanced storage capacities and faster hydrogen uptake and release rates compared to bulk metal hydrides [88, 89]. Nanomaterials further contribute to safety by addressing hydrogen's flammability concerns. Metal nanoparticles and carbon nanotubes emerge as candidates for hydrogen sensors, providing early warnings of potential safety hazards through their high sensitivity and selectivity to hydrogen gas [90].

### Reinforcing hydrogen pipelines

Hydrogen pipelines face challenges such as embrittlement due to the diffusion of hydrogen molecules into pipeline materials, causing cracking and damage. Nanomaterials, including graphene and carbon nanotubes, are under scrutiny as coatings for pipeline materials to enhance resistance to hydrogen embrittlement [91–94].

### Sustainability enhancement

Nanomaterials contribute not only to efficiency and safety but also to the sustainability of hydrogen energy systems. Utilizing nanomaterials for hydrogen production through renewable sources, such as solar and wind power, represents a promising avenue. Nanomaterials like titanium dioxide and iron oxide act as photocatalysts for hydrogen production through water splitting, leveraging their ability to absorb light energy and drive efficient chemical reactions [81, 86].

Table 5 provides a comprehensive overview of potential applications of nanomaterials in hydrogen transport and delivery, specifically in hydrogen storage tanks and pipelines. Metal–organic frameworks and carbon nanotubes exhibit promise for high-capacity hydrogen storage, while graphene demonstrates potential in improving hydrogen adsorption and desorption kinetics. Metal nanoparticles and metal–organic frameworks hold potential for promoting hydrogen dissociation and transport in pipelines, thus enhancing efficiency and reducing energy requirements. Table 5, highlights the potential applications of nanomaterials in hydrogen transport and delivery, specifically in hydrogen storage tanks and pipelines. Metal–organic frameworks and carbon nanotubes show promise for high-capacity hydrogen storage in tanks, while graphene can improve hydrogen adsorption and desorption kinetics. Metal nanoparticles and metal–organic frameworks have potential for promoting hydrogen dissociation and transport in pipelines, improving efficiency and reducing energy requirements. By utilizing the unique properties of nanomaterials, researchers can develop more efficient and cost-effective hydrogen transport and delivery technologies.

**Table 5:** Applications of Nanomaterials in Hydrogen Transport and Delivery

Application	Nanomaterial	Advantages
Hydrogen storage tanks	Metal–organic frameworks	High storage capacity and selectivity, potential for low-cost production and scalability, ability to be tailored for specific applications
Hydrogen storage tanks	Carbon nanotubes	High mechanical strength and stiffness, low density for lightweight storage, potential for enhanced safety and durability
Hydrogen storage tanks	Graphene	High electrical conductivity and surface area, potential for improved hydrogen adsorption and desorption kinetics
Hydrogen pipelines	Metal nanoparticles	High catalytic activity for promoting hydrogen dissociation and recombination, ability to improve pipeline efficiency and reduce energy requirements
Hydrogen pipelines	Metal–organic frameworks	High selectivity for hydrogen transport, potential for improved pipeline safety and durability, ability to be tailored for specific applications

This table provides an overview of how nanomaterials such as metal–organic frameworks, carbon nanotubes, graphene, and metal nanoparticles can be used to improve hydrogen transport and delivery, specifically in hydrogen storage tanks and pipelines. Understanding the potential advantages of these nanomaterials is crucial for developing practical applications of hydrogen technologies that can support a transition towards a more sustainable energy system. Overall, the use of nanomaterials in hydrogen transport and delivery represents a promising area of research with significant potential for improving the efficiency, safety, and sustainability of hydrogen energy systems. While there are still many challenges to overcome in terms of improving the scalability and practicality of nanomaterial-based hydrogen storage and transport systems, the ongoing research and development efforts in this field hold great promise for the future of clean energy.

### Future prospects and conclusion

The exploration of nanomaterials in hydrogen transport and delivery unfolds as a promising area of research with substantial potential to improve the efficiency, safety, and sustainability of hydrogen energy systems. Though challenges remain in terms of scalability and practicality, ongoing research and development efforts in this field instill confidence in a cleaner and more sustainable energy future.

## Current state of research and future prospects

### Recent advances in nanomaterial-based hydrogen technologies

The dynamic field of hydrogen energy and nanomaterials continuously witnesses breakthroughs and advancements. Notably, recent years have seen substantial progress in leveraging nanomaterials for hydrogen storage, production, and fuel cell efficiency. A particularly promising avenue is the application of metal–organic frameworks (MOFs) for hydrogen storage. MOFs, characterized by a porous structure composed of metal ions and organic ligands, exhibit high-pressure, low-temperature hydrogen adsorption capabilities. In 2020, Northwestern University researchers reported a novel MOF surpassing current materials in hydrogen storage efficiency [95–100].

In fuel cell research, exploration extends beyond Fe–N-C nanoparticles, encompassing other metal-based nanocatalysts like cobalt, nickel, and palladium. UCLA researchers, in 2019, introduced a cobalt–nickel nanocatalyst demonstrating superior performance compared to conventional platinum-based catalysts [101, 102]. Nanomaterials are also at the forefront of hydrogen production innovation. A catalyst composed of molybdenum sulfide nanosheets, developed in 2020 at the University of Illinois at Urbana-Champaign, efficiently splits water into hydrogen and oxygen, offering a potential leap in cost-effective hydrogen production methods [103, 104].

Active research into nanomaterials for enhancing the safety and efficiency of hydrogen storage tanks and pipelines is underway. The University of Central Florida's development of a composite material, combining carbon nanotubes and polyethylene, stands as a testament to ongoing efforts to bolster the strength and durability of hydrogen storage tanks [91–94].

### Future research directions and emerging trends

As we look to the future, numerous avenues beckon for further exploration in hydrogen energy and nanomaterials. Advancements in hydrogen fuel cell efficiency remain a pivotal area of interest, with researchers striving to develop new nanomaterials to enhance performance. Additionally, exploration into novel methods for hydrogen production and storage is underway, encompassing the use of MOFs and other porous materials [105, 106].

Table 6 provides a succinct summary of the current state of research in hydrogen energy, recent developments, and future prospects, categorically detailing the progress, recent breakthroughs, and anticipated trajectories in hydrogen production, storage, transport, and applications.

**Table 6:** Hydrogen energy: recent developments and future prospects

Category	Current State of research	Recent developments	Future prospects
Production	Focus on renewable sources, electrolysis, and processes	Decrease in hydrogen production cost from solar and wind sources	Scaling up green hydrogen production using renewable energy
Storage	Research on safe and efficient methods	New materials (e.g., MOFs, hydrides) for increased efficiency	Improvement in efficiency and safety of existing storage methods
Transport	Development and testing of hydrogen fuel cell vehicles	Global investments in hydrogen fuel cell technology and infrastructure	Widespread adoption hinges on infrastructure development and technology advancements
Applications	Various applications including transportation, heating, and power generation	Hydrogen as feedstock for ammonia production to reduce carbon footprint in fertilizer industry	Continued technology development for low-cost hydrogen availability and reduced carbon emissions

In conclusion, the intersection of nanomaterials and hydrogen energy emerges as a promising frontier, holding significant potential for enhancing the efficiency and sustainability of our energy systems. With ongoing research and development, nanomaterials are poised to play a crucial role in revolutionizing the production, storage, and transport of hydrogen in the years ahead.

## Expected economic importance

### Analysis of the potential economic impact of adopting nanomaterials in hydrogen technologies

Hydrogen energy, derived from renewable sources like wind and solar power, holds promise as a clean and sustainable energy source. However, efficient methods for hydrogen production, storage, and transport are vital for widespread adoption. Nanomaterials, with their unique properties, stand as catalysts for advancements in the hydrogen energy cycle. Their role in efficient catalysts for hydrogen production and high-capacity storage materials for fuel cells positions them at the forefront of transformative change. The development and commercialization of nanomaterials for hydrogen applications necessitate substantial research and development investments. The economic benefits, however, are substantial.

According to Allied Market Research, the global hydrogen energy market is poised to reach $201.2 billion by 2028, reflecting a 6.2% Compound Annual Growth Rate (CAGR) from 2021 to 2028. The escalating demand for clean and sustainable energy propels this growth, indicating a shift toward hydrogen energy solutions [107]. The integration of nanomaterials in advancing hydrogen energy has broader economic implications, spanning job creation and overall economic growth. The development of new technologies typically requires a skilled workforce, and the burgeoning hydrogen energy industry is expected to generate employment opportunities in research, development, manufacturing, and related sectors.

### Discussion on scalability, cost-effectiveness, and environmental sustainability

The economic benefits of nanomaterials in hydrogen energy extend beyond market growth. Job creation is a pivotal facet, and estimates suggest that the hydrogen industry could generate up to 700,000 new jobs in the United States alone by 2030. This encompasses roles in hydrogen production, transportation, storage, and fuel cell manufacturing, fostering employment opportunities across the value chain.

Reduced emissions emerge as a significant economic benefit. Hydrogen, being a clean-burning fuel, produces only water vapor and heat during combustion, presenting an environmentally attractive alternative to fossil fuels. By incorporating hydrogen into the energy mix, substantial reductions in greenhouse gas emissions can be achieved, contributing to improved air quality and mitigating climate change.

Enhancing energy security is another economic boon. Hydrogen production can utilize various domestic resources, including natural gas, coal, biomass, and water. This diversification minimizes dependence on fossil fuel imports, bolstering energy security for countries with limited reserves or high import dependency.

Job creation within the hydrogen economy is forecasted to have a considerable impact. The industry's growth is anticipated to create employment opportunities across various sectors, from production to transportation and fuel cell manufacturing. Estimates suggest that the hydrogen industry could generate up to 700,000 new jobs in the United States alone by 2030.

Increased efficiency is a crucial economic advantage of hydrogen fuel cells. Compared to internal combustion engines, hydrogen fuel cells exhibit higher efficiency, producing more power with less fuel. This heightened efficiency translates into cost savings and improved overall energy efficiency, further solidifying the economic viability of hydrogen technologies.

Energy storage capabilities amplify the economic benefits. Hydrogen, when stored, serves as a backup power source during peak demand or periods when renewable energy sources are unavailable. This contributes to grid stability and reduces the reliance on expensive energy storage systems, offering economic advantages alongside enhanced reliability.

Cost reduction is a pivotal factor in the economic feasibility of hydrogen energy. As technology evolves for hydrogen production, storage, and transportation, costs are expected to decrease. This anticipated reduction positions hydrogen as a cost-effective alternative to fossil fuels in the long term, presenting economic advantages on a global scale.

In conclusion, the economic importance of incorporating nanomaterials in advancing hydrogen energy is multifaceted. Beyond market growth, the potential for job creation, increased efficiency, enhanced energy security, and environmental sustainability underscores the transformative impact of nanomaterials in shaping a sustainable and economically robust energy future. Table 7, summarizing the expected economic benefits of hydrogen energy, including reduced emissions, increased energy security, job creation, increased efficiency, energy storage, and reduced costs.

**Table 7:** Expected Economic Benefits of Hydrogen Energy

Economic benefit	Description
Reduced emissions	Hydrogen is a clean-burning fuel that produces only water vapor and heat when it is burned. This makes it an attractive alternative to fossil fuels, which produce harmful emissions such as carbon dioxide, nitrogen oxides, and particulate matter. By using hydrogen as an energy source, we can significantly reduce greenhouse gas emissions and improve air quality
Energy security	Hydrogen can be produced from a variety of domestic resources, including natural gas, coal, biomass, and water. This means that countries that have limited fossil fuel reserves or are dependent on imports can use hydrogen as a way to increase their energy security and reduce their dependence on foreign oil
Job creation	The development of a hydrogen economy is expected to create new jobs in areas such as hydrogen production, transportation, storage, and fuel cell manufacturing. According to some estimates, the hydrogen industry could create up to 700,000 new jobs in the United States alone by 2030
Increased efficiency	Hydrogen fuel cells are more efficient than internal combustion engines, meaning that they can produce more power with less fuel. This can help to reduce the overall energy consumption of a system, resulting in cost savings and improved energy efficiency
Energy storage	Hydrogen can be stored for long periods of time and used as a backup power source during times of high demand or when renewable energy sources are not available. This can help to stabilize the electricity grid and reduce the need for expensive energy storage systems
Reduced costs	As the technology for producing, storing, and transporting hydrogen improves, the cost of using hydrogen as an energy source is expected to decrease. This could make hydrogen a more cost-effective alternative to fossil fuels in the long term

## Conclusion

In conclusion, nanomaterials have tremendous potential for advancing the hydrogen energy frontier. Their unique properties, such as high surface area, tunable surface chemistry, and high catalytic activity, make them attractive candidates for various hydrogen-related applications, including hydrogen storage, production, and transport. One of the main advantages of using nanomaterials in hydrogen-related applications is their ability to improve efficiency and reduce costs. For example, using nanomaterials as catalysts in hydrogen fuel cells can reduce the amount of expensive platinum required, making fuel cell technology more economically viable. Similarly, using nanomaterials for hydrogen storage can increase storage capacity while reducing the weight and volume of storage tanks, making it easier and more cost-effective to transport and store hydrogen. Despite the many benefits of using nanomaterials in hydrogen-related applications, several challenges still need to be overcome. For example, there is a need to develop scalable and cost-effective methods for synthesizing and processing nanomaterials. There is also a need to improve our understanding of the properties and behavior of nanomaterials at the nanoscale, as well as their interaction with other materials and the environment. Future research directions in this field should focus on developing new nanomaterials with enhanced properties and functionalities for hydrogen-related applications. For example, the use of 2D materials, such as graphene and transition metal dichalcogenides, is a promising area of research that has shown potential for improving the performance of hydrogen storage and production. In addition, the development of nanomaterials that can withstand harsh operating conditions, such as high temperature and pressure, is also an important area of research. Another important direction for future research is to develop methods for integrating nanomaterials into practical hydrogen-related devices and systems. This includes the development of scalable and cost-effective methods for producing nanomaterials, as well as the design and optimization of nanomaterial-based devices and systems.

In conclusion, the use of nanomaterials for advancing the hydrogen energy frontier is an exciting and promising area of research that has the potential to revolutionize the energy industry. While there are still many challenges to be overcome, the development of new and improved nanomaterials for hydrogen-related applications offers tremendous opportunities for improving efficiency, reducing costs, and creating a sustainable energy future.
